# Higher Daily Physical Activity Level Is Associated with Lower RBC Aggregation in Carotid Artery Disease Patients at High Risk of Stroke

**DOI:** 10.3389/fphys.2017.01043

**Published:** 2017-12-12

**Authors:** Pauline Mury, Camille Faes, Antoine Millon, Mathilde Mura, Céline Renoux, Sarah Skinner, Virginie Nicaise, Philippe Joly, Nellie Della Schiava, Patrick Lermusiaux, Philippe Connes, Vincent Pialoux

**Affiliations:** ^1^Interuniversity Laboratory of Human Movement Biology EA7424, Vascular Biology and Red Blood Cell Team, University Claude Bernard Lyon 1, Villeurbanne, France; ^2^Laboratory of Excellence GR-Ex, Paris, France; ^3^CarMeN Laboratory, Institut National de la Santé et de la Recherche Médicale U1060, University Claude Bernard Lyon 1, Bron, France; ^4^Department of Vascular Surgery, Edouard Herriot Hospital, Lyon, France; ^5^Biochimie des Pathologies Erythrocytaires, Centre de Biologie et de Pathologie Est, Hospices Civils de Lyon, Lyon, France; ^6^Laboratory of Vulnerabilities and Innovation in Sport EA7428, University Claude Bernard Lyon 1, Villeurbanne, France; ^7^Institut Universitaire de France, Paris, France

**Keywords:** atherosclerosis, blood rheology, chronic physical activity, red blood cell aggregation, stroke

## Abstract

**Aim:** Carotid artery disease (CAD) is an atherosclerotic inflammatory disease that affects the arterial wall, specifically at points of bifurcation where blood flow is disturbed. Abnormal blood rheology could participate in the pathophysiology of ischemic cardiovascular disease. Physical activity (PA) is known to improve blood rheology in several chronic disorders. This study aims to (i) compare the hemorheological profile of CAD patients and controls and (ii) investigate the associations between daily PA and hemorheological parameters in CAD patients.

**Methods:** Blood viscosity, red blood cell (RBC) aggregation and RBC deformability were assessed in 80 patients (15 symptomatic and 65 asymptomatic) and 14 age-matched controls. Patients' PA levels were evaluated using questionnaires.

**Results:** Symptomatic patients showed increased blood viscosity and RBC aggregation compared to healthy controls. RBC aggregation was significantly lower in the most physically active patients compared to the least physically active ones. Blood viscosity and RBC deformability did not vary according to physical activity level.

**Conclusions:** Our results showed greater hemorheological abnormalities (blood hyper-viscosity and hyper-aggregation of red blood cells) in the most severe CAD patients, which could exacerbate the risk of stroke in patients with stenosis. As the most physically active patients had lower RBC aggregation than those who were less physically active, it is possible that regular PA may limit hemorheological alterations in CAD patients.

## Introduction

Atherosclerosis is the leading cause of mortality and morbidity in western countries, and will likely become the leading cause of death worldwide in the near future. Atherosclerosis is a complex inflammatory disease that affects the arterial wall, leading to atherosclerotic plaque formation (Redgrave et al., 2008). Atherosclerotic plaques build up in specific regions of the arterial system, such as in carotid bifurcations, the aortic arch, and in the femoral arteries. In these regions, blood flow is disturbed and the expression of biomarkers of atherogenesis and thrombosis correlates with the extent of flow recirculation patterns (Martorell et al., 2014). In atherosclerotic patients carotid plaque rupture can lead to major ischemic events, such as stroke and transient ischemic attack (TIA) (Redgrave et al., 2008).

Over the last two decades, several studies have investigated the role of blood rheology in cardiovascular diseases, particularly in ischemic disease (Wood and Kee, 1985; Fisher and Meiselman, 1991). Blood viscosity represents the resistance of blood to flow and may be affected by the proportion of red blood cells (RBC) in the blood (i.e., hematocrit), as well as by the rheological properties of these RBCs: i.e., RBC deformability and RBC aggregation (Baskurt and Meiselman, 2003). RBC aggregation is a reversible process, which affects blood viscosity at low shear rate. In contrast, RBC deformability influences blood viscosity mainly at high shear rate. When RBC deformability decreases, RBCs loose their ability to properly orient themselves in the direction of the blood flow, thereby increasing blood viscosity. Nevertheless, RBC aggregation has been shown to affect blood flow in both micro- and macro-vessels where shear rate is high, notably because of its effects on several rheological phenomena such as axial migration, cell free layer formation, and the Fahraeus effect, the latter occurring mainly in the microcirculation (Baskurt and Meiselman, 2008). Recently, it was shown that patients with symptomatic ischemic cerebral events had increased blood viscosity in comparison to asymptomatic patients (Li et al., 2015; Totsimon et al., 2016) and healthy controls (Velcheva et al., 2006). Lower RBC deformability could be at the origin of this blood hyper-viscosity (Totsimon et al., 2016). Research also suggests that RBC aggregation could play a role in ischemic cerebral disease (Zeltser et al., 2001). Interestingly, Assayag et al. reported an association between increased RBC aggregation and the degree of stenosis in patients with asymptomatic carotid artery disease (Assayag et al., 2008). Indeed, abnormal blood rheology could be involved in the pathophysiology of ischemic stroke.

Regular physical activity (PA) has been shown to have beneficial effects on atherosclerosis, particularly in preventing adverse cardiovascular events (Sofi et al., 2008) and reducing the incidence of carotid artery disease (Stein et al., 2015). Regular PA has been shown to reduce cardiovascular risk factors, such as blood lipids, cholesterol, and body mass index (BMI) (Taylor et al., 2014). In addition, regular PA could have beneficial effects on blood rheology in healthy subjects and patients with cardiovascular or metabolic diseases (Connes et al., 2013). For example, Sandor et al. demonstrated a reduction in blood viscosity after 12 and 24 weeks of moderate aerobic exercise training in ischemic heart disease patients (Sandor et al., 2014). This reduction was attributed to decreased RBC aggregation and improved RBC deformability (Sandor et al., 2014). However, it is currently unknown whether high levels of PA, defined as 300 min or more of moderate-intensity PA per a week, (compared to moderate levels of PA, defined as less than 150 min of moderate-intensity PA per week) could limit, or improve, blood rheological alterations in patients at high risk of ischemic stroke, as it has been shown to do in cardiovascular disease patients.

The aim of the present study was (i) to compare the blood rheological profile of symptomatic and asymptomatic patients at high-risk of stroke who underwent endarterectomy surgery with age-matched healthy controls and (ii) to evaluate the associations between daily PA level and blood rheology in these patients. We hypothesized that symptomatic patients would present more blood rheological abnormalities than asymptomatic patients, and that PA could limit these alterations. Questionnaires, such as the GPAQ, are a simple, safe, valid, and reliable way to initially evaluate patients' PA levels, which could easily be used in a clinical setting.

## Patients and methods

### Patients

Eighty patients (67 men and 13 women of 68.7 ± 10.8 years) undergoing carotid endarterectomy in the vascular surgery department of Edouard Herriot Hospital of Lyon were included in this study. Among them, 15 were symptomatic (i.e., patients who had a history of ischemic events, including ischemic stroke or transient ischemic attack) and 65 were asymptomatic (i.e., patients with no history of ischemic events). All symptomatic and asymptomatic CAD patients underwent unilateral carotid endarterectomy, except for three patients who underwent two surgeries at a 4–6 weeks interval. Carotid stenosis was measured and quantified by Doppler ultrasound and confirmed by either magnetic resonance or computed tomography angiography 1–6 months before the surgery. Six patients also presented with bilateral carotid stenosis, but were only operated on one side. All patients were treated by statins, platelet anti-aggregants and angiotensin converting enzyme (ACE) inhibitors, as recommended by the French national guidelines (Haute Autorité de Santé, 2015) for patients with planned endarterectomy. Within 30 min before surgery (just before anesthesia), venous blood samples (2 × 7 ml) were drawn from the cephalic vein in tubes containing ethylenediamine tetraacetic acid (EDTA). Patients were in a fasted state. Written informed consent for the analysis of blood was obtained from all patients before surgery. This study was approved by the local ethics committee. Main cardiovascular risk factors, including hypertension, dyslipidemia, diabetes mellitus, smoking status, and body mass index (BMI), were recorded by the anesthetist. Fourteen age-matched healthy persons were also included as controls (63.6 ± 13.0 years, 6 men and 8 women) for blood rheological measurements only, with blood sampled in the fasted state.

### Questionnaires

Among the 80 patients included in this study, only 68 answered questionnaires (12 patients did not respond). All patients responded to two questionnaires by phone. The Mini Mental State Examination (MMSE) questionnaire was used to confirm their mental health state after the surgery, and the Global Physical Activity Questionnaire (GPAQ) was used to measure the recreational PA levels of 68 of the 80 patients (13 symptomatic patients and 55 asymptomatic patients).

The GPAQ was originally designed by the World Health Organization (WHO) to assess PA patterns in western countries (Bull et al., 2009). The data in this study were analyzed according to the GPAQ Analysis Guide provided by the WHO (retrieved 2015). Recreational PA was expressed in min/day. For data analysis, all patients (symptomatic and asymptomatic) in the cohort were ranked in increasing order of duration of daily PA, and divided by 3 to obtain three PA tertiles corresponding to duration of daily PA (T1: 3.3 ± 0.9 min/day, T2: 27.98 ± 1.89 min/day and T3: 109.9 ± 10.18 min/day for the less, intermediate and the more active, respectively).

### Biological parameters and blood rheology

Hemoglobin (Hb) concentration, RBC, white blood cell (WBC), platelet, neutrophil, lymphocyte, and monocyte counts of patients were determined using a hematology analyzer (Coulter LH 750, Beckman Coulter, CA, USA). Glucose levels were determined using an automated immunoassay analyzer (Dimension Vista 1500, Siemens, UK).

Blood rheological parameters were measured after full blood oxygenation and within 4 h after sampling in patients and healthy controls. Blood viscosity was measured at native hematocrit (Hct) using a cone/plate viscometer (Brookfield DVII+ with CPE40 spindle; Brookfield Engineering Labs, Natick, MA) at 225 s^−1^ to mimic the shear rates usually found in large arteries such as the carotid artery (Baskurt et al., 2009; Connes et al., 2013). RBC deformability, reported as an elongation index, was determined at 37°C and 9.49 Pa (LORRCA MaxSis; RR Mechatronics, Hoorn, The Netherlands). RBC aggregation, reported as an aggregation index (Hardeman et al., 2001), was determined at 37°C by syllectometry (LORRCA MaxSis; Mechatronics, The Netherlands) after adjustment of Hct to 40%. The RBC disaggregation threshold (γ), the minimal shear rate needed to prevent aggregation or to break down pre-existing RBC aggregates, was determined using a reiteration procedure. The system has been described elsewhere in detail (Baskurt et al., 2009).

Plasma fibrinogen was measured by the Clauss method using an ACL Top automatizer (Werfen, Barcelona, Spain). The Clauss method determines a thrombin time based on optical detection of fibrin formation against a standard curve from a referent plasma sample. The tests were part of the standard clinical care of patients undergoing endarterectomy surgery.

### Statistical analysis

Biological results are expressed as mean ± SEM. A nonparametric Mann-Whitney test was used to compare biological parameters between symptomatic and asymptomatic patients. A nonparametric Kruskal-Wallis test followed by Dunn *post-hoc's* test was used to compare biological and clinical parameters between PA level tertiles.

Spearman correlation was used to test the relationship between the hemorheological parameters and the percent stenosis, and between the hemorheological parameters and the PA level. A linear multivariate model was used to test the independent predictors of blood viscosity and RBC aggregation. Significance level was defined as *p* < 0.05. Analyses were conducted using GraphPad Prism software (v. 6, San Diego, CA, USA).

## Results

### General characteristics and hematological parameters

Age, cardiovascular risk factors (hypertension, dyslipidemia, diabetes mellitus, smoking, BMI) and biological parameters (RBC, WBC, Hb, platelets, neutrophils, lymphocytes, monocytes, glucose) were not significantly different between symptomatic patients and asymptomatic patients (Table 1).

**Table 1:** Cardiovascular risk factors and biological characteristics in the whole population.

	**Symptomatic patients (*n* = 15)**	**Asymptomatic patients (*n* = 65)**	**Reference values**
Men/Women	12/3	55/10	
Age (years)	69.0 ± 12.6	68.4 ± 9.1	
Hypertension	9 (60%)	43 (66.2%)	
Dyslipidemia	8 (53.3%)	22 (33.8%)	
Diabetes mellitus	2 (15.3%)	19 (29.2%)	
Smoking			
Active	1 (6.67%)	6 (9.23%)	
Weaned	8 (53.3%)	39 (60%)	
Pack-year	45.0 ± 8.16	31.9 ± 3.34	
BMI	27.5 ± 1.38	26.0 ± 0.46	
Percent stenosis	57.5 ± 4.36	69.53 ± 2.53^**^	
RBC (10^−12^/L)	4.84 ± 0.17	4.55 ± 0.06	4.5–6
WBC (10^−9^/L)	8.03 ± 0.95	7.85 ± 0.28	4.0–10.0
Hb (g/L)	134.0 ± 4.61	136.8 ± 1.86	130–170
Platelets (10^−9^/L)	238.1 ± 14.78	234.3 ± 7.79	150–400
Neutrophils (10^−9^/L)	5.50 ± 0.85	5.07 ± 0.23	1.8–7.5
Lymphocytes (10^−9^/L)	1.74 ± 0.19	1.96 ± 0.09	1.0–4.0
Monocytes (10^−9^/L)	0.57 ± 0.07	0.65 ± 0.03	0.2–0.9
Glucose (mmol/L)	5.90 ± 0.31	6.64 ± 0.36	4.1–5.8

Values are expressed as mean ± SEM, and percent within the group. BMI, body mass index; RBC, red blood cell count, WBC, white blood cell count, Hb, hemoglobin.

** *p < 0.01 significant difference compared to symptomatic patients*.

### Blood rheological parameters in symptomatic/asymptomatic patients

**Figure 1:**
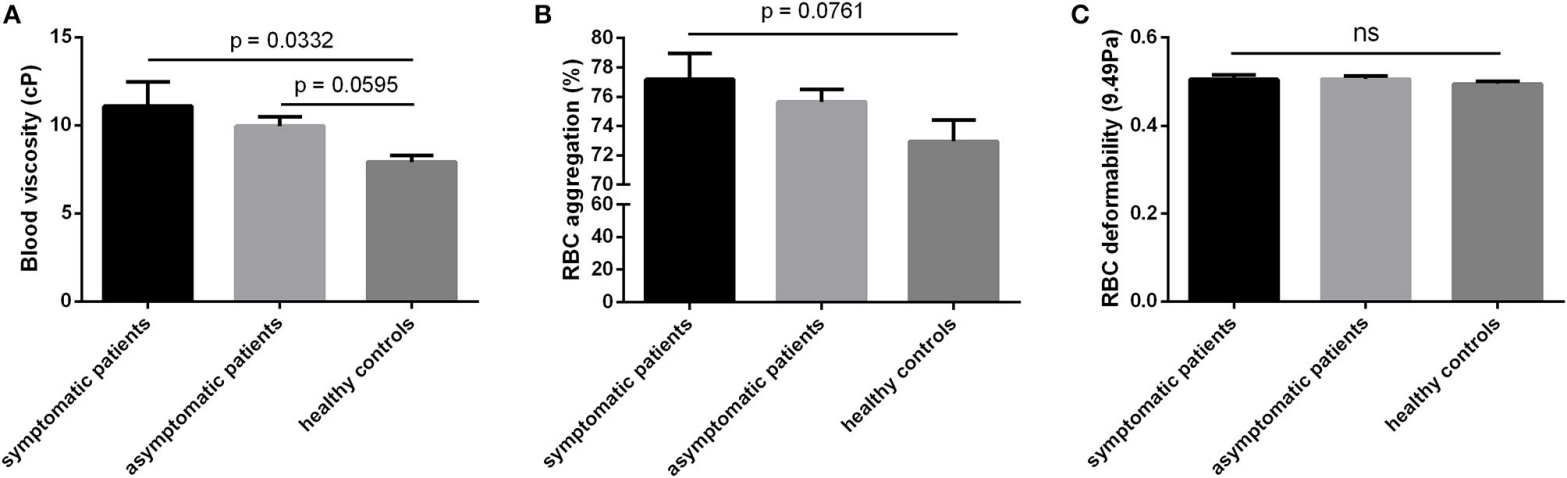
Hemorheological parameters of symptomatic patients, asymptomatic patients at high-risk of stroke and healthy controls. **(A)** Blood viscosity in cP, **(B)** RBC aggregation in % and **(C)** RBC deformability at 9.49Pa. Values are expressed as mean ± SEM.

Blood viscosity was significantly higher in symptomatic patients (*p* < 0.05) compared to healthy individuals and a trend (*p* = 0.0595) for higher blood viscosity in asymptomatic patients compared to healthy controls was also observed (Figure 1A).

No significant differences were observed between the groups for RBC aggregation (Figure 1B). Nevertheless, RBC aggregation tended to be higher in symptomatic compared to healthy controls (*p* = 0.0761). Moreover, RBC aggregation was positively correlated with the percent of stenosis (*p* < 0.05; *r* = 0.889) in symptomatic patients. RBC deformability (Figure 1C), Hct, fibrinogen level, and RBC aggregates strength did not differ between the three groups (Table 2). In addition, although the dosage of statins, platelet antiaggregants, and ACE inhibitors may differ between subjects, we did not find any significant correlations between the dose of these drugs and blood rheological parameters (data not shown).

**Table 2:** Hemorheological parameters in symptomatic patients, asymptomatic patients at high-risk of stroke and healthy controls.

	**Symptomatic patients (*n* = 15)**	**Asymptomatic patients (*n* = 65)**	**Healthy controls (*n* = 14)**
RBC aggregates strength (s^−1^)	310.8 ± 63.6	319.0 ± 28.03	254.5 ± 24.82
Hct (%)	40.5 ± 0.84	41.7 ± 0.63	41.1 ± 0.47
Fibrinogen (g/L)	3.85 ± 0.25	3.52 ± 0.14	3.54 ± 0.24

*Values are expressed as mean ± SEM*.

### Blood rheological parameters and daily physical activity effects

No differences in age or in cardiovascular risk factors (hypertension, dyslipidemia, diabetes mellitus, smoking, BMI) were found between the patients' subgroups, which were composed according to their daily PA level. Higher WBC (9.03 ± 0.82 vs. 6.79 ± 0.38 10^−9^ /L, respectively, *p* < 0.05), neutrophil (6.39 ± 0.74 vs. 4.19 ± 0.29 10^−9^/L, respectively, *p* < 0.01) and platelet counts (260.7 ± 17.76 vs. 208.0 ± 8.41 10^−9^/L, respectively, *p* < 0.05) were found in the less physically active patients (T1) compared to the most physically active ones (T3). Monocytes tended to be higher in T1 than in T3 (*p* = 0.0597). Nevertheless, WBC, neutrophil, platelet, and monocyte counts remained in the normal range of values across the three groups. No other differences in biological parameters (RBC, Hb, lymphocytes, glucose) were found between tertiles. General characteristics regarding the tertiles are reported in Table 3.

**Table 3:** Cardiovascular risk factors and biological characteristics of the different subgroups divided according to their PA level.

	**Physical activity T1 (*n* = 21)**	**Physical activity T2 (*n* = 23)**	**Physical activity T3 (*n* = 24)**	**References values**
Men/Women	16/5	21/2	19/5	
Symptomatic/asymptomatic	5/16	4/19	4/20	
Age (years)	70.1 ± 2.0	67.7 ± 2.1	68.5 ± 1.9	
Hypertension	14 (66.7%)	16 (69.5%)	17 (70.8%)	
Dyslipidemia	7 (33.3%)	11 (47.8%)	8 (33.3%)	
Diabetes mellitus	7 (33.3%)	5 (21.7%)	5 (20.8%)	
Smoking				
Active	4 (19.0%)	1 (4.35%)	2 (8.33%)	
Weaned	14 (66.7%)	17 (73.9%)	14 (58.3%)	
Pack-year	22.5 ± 7.89	33.3 ± 7.53	25.8 ± 5.47	
BMI	26.7 ± 0.97	26.7 ± 0.93	25.7 ± 0.81	
Percent stenosis	66.67 ± 4.17	70.54 ± 2.76	67.67 ± 3.41	
RBC (10^−12^/L)	4.47 ± 0.13	4.57 ± 0.11	4.50 ± 0.10	4.5–6
WBC (10^−9^/L)	9.03 ± 0.82	8.07 ± 0.41	6.79 ± 0.38^*^	4.0–10.0
Hb (g/L)	134.2 ± 3.88	139.5 ± 2.80	135.0 ± 3.31	130–170
Platelets (10^−9^/L)	260.7 ± 17.76	241.3 ± 10.38	208.0 ± 8.41^*^	150–400
Neutrophils (10^−9^/L)	6.39 ± 0.74	5.30 ± 0.34	4.19 ± 0.29^**^	1.8–7.5
Lymphocytes (10^−9^/L)	1.91 ± 0.17	1.91 ± 0.14	1.80 ± 0.16	1.0–4.0
Monocytes (10^−9^/L)	0.72 ± 0.08	0.68 ± 0.05	0.55 ± 0.03	0.2–0.9
Glucose (mmol/L)	6.19 ± 0.53	6.46 ± 0.46	6.43 ± 0.52	4.1–5.8

Tertiles; T1, the less physically active patients (3.3 ± 0.9 min/day), T2, the intermediate physically active patients (27.98 ± 1.89 min/day), T3, the most physically active patients (109.9 ± 10.18 min/day). Values are expressed as mean ± SEM, and percent within the group. BMI, body mass index; RBC, red blood cell count, WBC, white blood cell count, Hb, hemoglobin.

* p < 0.05

** *p < 0.01 significant difference compared to T1*.

**Figure 2:**
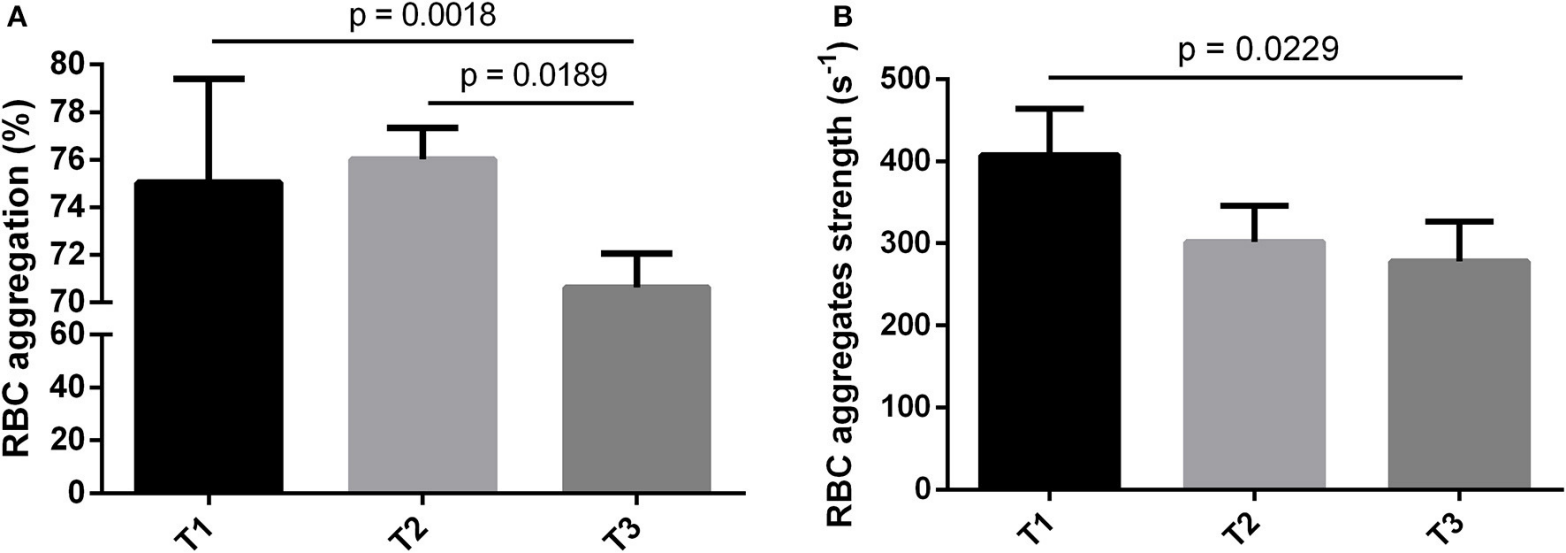
Hemorheological parameters of the different subgroups divided according to their PA level (T = Tertiles). **(A)** RBC aggregation in %, **(B)** RBC aggregates strength in s^−1^. Values are expressed as mean ± SEM. T1: the less physically active patients (3.3 ± 0.9 min/day), T2: the intermediate physically active patients (27.98 ± 1.89 min/day), T3: the most physically active patients (109.9 ± 10.18 min/day).

RBC aggregation was significantly higher (*p* < 0.01) in T1 compared to T3 (*p* < 0.01) and in T2 compared to T3 (*p* < 0.05) (Figure 2A), but did not differ between T1 and T2 (Figure 2A). RBC aggregation was negatively correlated with PA level (*p* < 0.001; *r* = −0.4423) for all patients (symptomatic and asymptomatic).

RBC aggregates strength (i.e., the force needed to dissociate the RBC aggregates; also called the RBC disaggregation threshold) was significantly higher (*p* < 0.05) in T1 compared to T3 (Figure 2B). No difference in RBC aggregates strength was observed between T1 and T2 or T2 and T3. RBC aggregates strength was negatively correlated with the PA level (*p* < 0.01; *r* = −0.3466) in all patients (symptomatic and asymptomatic). The other hemorheological parameters did not differ between the tertiles (Table 4).

**Table 4:** Hemorheological parameters of the different subgroups divided according to their PA level.

	**Physical activity T1 (*n* = 21)**	**Physical activity T2 (*n* = 23)**	**Physical activity T3 (*n* = 24)**
Blood viscosity (cP)	9.35 ± 0.81	10.75 ± 0.84	8.95 ± 0.49
RBC deformability (9,49Pa, a.u.)	0.439 ± 0.016	0.454 ± 0.006	0.449 ± 0.009
Hct (%)	40.8 ± 1.18	42.9 ± 1.01	40.7 ± 0.93
Fibrinogen (g/L)	3.87 ± 0.28	3.59 ± 0.22	3.34 ± 0.18

*T1, the less physically active patients (3.3 ± 0.9 min/day); T2, the intermediate physically active patients (27.98 ± 1.89 min/day); T3, the most physically active patients (109.9 ± 10.18 min/day). Values are expressed as mean ± SEM. A.u, arbitrary units*.

A linear multivariate model was used to test the independent association between all of the parameters listed in Table 3 and blood viscosity or RBC aggregation. For blood viscosity, the overall model was not significant (*R*2 = 0.31, *p* = 0.28). In contrast, the model was significant for RBC aggregation (*R*2 = 0.52, *p* = 0.05) with PA being the only factor independently associated with RBC aggregation.

## Discussion

Our results show that a higher daily physical activity level is associated with lower RBC aggregation and decreased RBC aggregates strength in patients at high-risk of stroke who underwent endarterectomy surgery. Moreover, RBC aggregation and blood hyperviscosity tended to be higher in the most symptomatic patients.

As previously described in the literature (Koenig and Ernst, 1992; Velcheva et al., 2006, 2010; Li et al., 2015) blood viscosity was significantly increased in carotid symptomatic patients in comparison to healthy subjects. We also found a tendency (*p* = 0.059) for asymptomatic patients to have higher blood viscosity than healthy subjects. As previously mentioned, blood viscosity is primarily determined by hematocrit, plasma viscosity, RBC deformability, and aggregation (Dintenfass, 1985). In our study, RBC deformability did not differ between symptomatic patients, asymptomatic patients, and healthy subjects, while RBC aggregation tended to be higher in symptomatic patients compared to healthy subjects. Although blood viscosity was measured at a high shear rate (225 s^−1^), it is possible that the presence of persistent RBC aggregates could have affected blood viscosity. Indeed, several studies have previously demonstrated the presence of persistent RBC aggregates in large arteries, where the shear rate is over 50–100 s^−1^ (Baskurt and Meiselman, 2008). In the present study, the mean RBC disaggregation threshold in both symptomatic and asymptomatic patients was around 300 s^−1^. Therefore, it is possible that the tendency for increased RBC aggregation observed in the symptomatic patient group, compared to the control group, could be partially responsible for the higher blood viscosity found in this group. A limitation of this study is that blood viscosity was not measured at a low shear rate (i.e., <1 s^−1^), when RBC aggregates are present in a large proportion.

Our study also showed a positive correlation between RBC aggregation and the percent of stenosis in symptomatic patients. It has previously been demonstrated that increased RBC aggregation enhances the axial migration of RBC, increases the width of the cell-free layer near the vascular wall, and promotes the migration of platelets and leucocytes to the endothelium, hence favoring their adhesion (Goldsmith et al., 1999; Baskurt et al., 2007). Increased cellular adhesion is clearly identified as a major risk factor for atherosclerosis and plaque formation (Galkina and Ley, 2007). Moreover, RBC adherence to the arterial wall may be increased at arterial bifurcations. The elevated amount of circulating, sticky RBC aggregates present in CAD could exacerbate this phenomenon, as has been previously demonstrated in sickle cell disease (Loiseau et al., 2015). The increased deposition of RBC aggregates into the vascular wall would then participate in the alteration of flow dynamics around the bifurcations and promote the deposition of other circulating cellular elements involved in atherogenesis.

Previous studies demonstrated a decrease in RBC aggregation after exercise training in healthy individuals and patients with cardiovascular or metabolic diseases (Dintenfass and Lake, 1976; Simmonds et al., 2012; Connes et al., 2013; Sandor et al., 2014; Pabisiak et al., 2015). However, to our knowledge this is the first time that interactions between daily PA level and blood rheology have been investigated in the context of patients at high risk of stroke. Our results support the hypothesis that PA in symptomatic and asymptomatic patients could improve RBC aggregation properties. Fibrinogen is one of the most pro-aggregant molecules in the plasma and can be affected by exercise training (Pabisiak et al., 2015). However, plasma fibrinogen concentration was not different between our three groups. RBC aggregation depends both on cellular (RBC aggregability) and plasma factors (Baskurt and Meiselman, 2009) and it could be hypothesized that regular PA could affect the intrinsic ability of RBC to form aggregates. Regular PA has also been shown to lower oxidative stress and inflammation, and limit the vascular adhesion process, all of which are known to be involved in the development and progression of atherosclerotic plaques (Rush et al., 2005; Pialoux et al., 2009; Szostak and Laurant, 2011). The anti-oxidant and anti-inflammatory effects of regular PA could also explain why the most active patients have decreased RBC aggregation. For example, Hierso et al. demonstrated that higher RBC oxidative stress increased the robustness of RBC aggregates (Hierso et al., 2014). Alternatively, decreased RBC aggregation and RBC aggregates strength could possibly be attributed to decreased platelet and leucocyte counts in the most active patients. Further studies are needed to better understand the mechanisms of the decreased RBC aggregation in physically active patients at risk of stroke.

The American Heart Association and the European Society of Cardiology have provided PA recommendations for adults to improve overall cardiovascular health and prevent cardiovascular diseases (Perk et al., 2013; Eckel et al., 2014). Currently, these organizations recommend that adults practice 30 min of moderate PA (at 70–85% of maximal heart rate), 3–5 times a week. Furthermore, it appears that long-term exercise training is more beneficial than short-term exercise training in improving cardiovascular morbidity and mortality prognosis (Giannuzzi et al., 2008; Wood et al., 2008). In our study, we assessed daily PA level, which can be used to extrapolate long-term PA. We observed patients whose daily PA surpassed the 30 min a day recommended by the European Society of Cardiology (respectively T3: 109.9 ± 10.18 vs. T1: 3.26 ± 0.89 and T2: 27.98 ± 1.89 min/day), presented with fewer rheological abnormalities.

One limitation of our study could be the relative subjectivity of the GPAQ responses, since in the general population (Laeremans et al., 2017) and in cancer patients (Ruiz-Casado et al., 2016) the GPAQ has been shown to slightly overestimate PA level when compared to accelerometer data. However the GPAQ is a standardized and validated questionnaire (Cleland et al., 2014). In this context, the overestimation reported in these studies (Ruiz-Casado et al., 2016; Laeremans et al., 2017) was related to PA intensity level (in METs) and not to the duration of PA per day, which is the criteria that we used to characterize our PA values. Furthermore, although PA questionnaires may overestimate energy expenditures, the PA thresholds reported in the literature corresponding to decreased cardiovascular risk have been confirmed using questionnaires, including the GPAQ, in large cohorts of subjects (World Health Organization, 2010; Kyu et al., 2016; Zhao et al., 2016; Poggio et al., 2017). Finally, the differences found between the three tertile groups (3.3 vs. 27.9 vs. 109.9 min/day) are sufficiently significant to distinguish the effect of PA duration on blood rheology parameters between the groups. Nevertheless, further studies using more objective cardiorespiratory and physical fitness measurements are needed to confirm the relationship between PA and blood rheology in CAD patients.

In conclusion, to the best of our knowledge, our study is the first to suggest that physically active CAD patients at high risk of stroke could present with fewer rheological abnormalities than those who practice little or no PA. This indicates that regular PA could possibly limit the hemorheological alterations generally observed in CAD patients at high risk of stroke, thereby minimizing the risk of carotid arterial stenosis and ischemic events in those patients. Further studies with objective measures of PA are needed to confirm the effect of PA on RBC aggregation in this population.

## Ethics statement

This study was carried out in accordance with the recommendations of the guidelines set by the Declaration of Helsinki with written informed consent from all subjects. All subjects gave written informed consent in accordance with the Declaration of Helsinki. The protocol was approved by the “CPP Sud-Est IV, Lyon, France.”

## Author contributions

PM, PC, and VP participated in design the study; PM, CR, MM, and VN performed the experiments; PM, AM, MM, ND, and PL included the patients; PM, CF, PC, and VP analyzed and interpreted the results; PM, CF, PC, and VP wrote the manuscript; PM, CF, AM, MM, CR, SS, VN, PJ, ND, PL, PC, and VP wrote and edited the manuscript.

### Conflict of interest statement

The authors declare that the research was conducted in the absence of any commercial or financial relationships that could be construed as a potential conflict of interest.
